# Oral *Candida* administration in a *Clostridium difficile* mouse model worsens disease severity but is attenuated by *Bifidobacterium*

**DOI:** 10.1371/journal.pone.0210798

**Published:** 2019-01-15

**Authors:** Wimonrat Panpetch, Naraporn Somboonna, Matanee Palasuk, Pratsanee Hiengrach, Malcolm Finkelman, Somying Tumwasorn, Asada Leelahavanichkul

**Affiliations:** 1 Department of Microbiology, Faculty of Medicine, Chulalongkorn University, Bangkok, Thailand; 2 Department of Microbiology, Faculty of Science, Chulalongkorn University, Bangkok, Thailand; 3 Associates of Cape Cod, Inc., East Falmouth, MA, USA; 4 Center of Excellence in Immunology and Immune-mediated Diseases, Department of Microbiology, Chulalongkorn University, Bangkok, Thailand; University of Bristol, UNITED KINGDOM

## Abstract

Gut fungi may influence the course of *Clostridium difficile* infection either positively or negatively for the host. Fungi are not prominent in the mouse gut, and *C*. *albicans*, the major human gastrointestinal commensal yeast, is in low abundance or absent in mice. *Bifidobacterium* is one of the probiotics that may attenuate the severity of *C*. *difficile* infection. Inflammatory synergy between *C*. *albicans* and *C*. *difficile*, in gut, may provide a state that more closely resembles human infection and be more suitable for testing probiotic effects. We performed fecal mycobiota analysis and administered *C*. *albicans* at 1 day prior to *C*. *difficile* dosing. Fecal eukaryotic 18S rDNA analysis demonstrated the presence of Ascomycota, specifically, *Candida* spp., after oral antibiotics, despite negative fecal fungal culture. *C*. *albicans* administration enhanced the severity of the *C*. *difficile* infection model as determined by mortality rate, weight loss, gut leakage (FITC-dextran assay), and serum and intestinal tissue cytokines. This occurred without increased fecal *C*. *difficile* or bacteremia, in comparison with *C*. *difficile* gavage alone. *Candida* lysate with *C*. *difficile* increased IL-8 production from HT-29 and Caco-2 human intestinal epithelial cell-lines. *Bifidobacterium* attenuated the disease severity of the *C*. *difficile* plus *Candida* model. The reduced severity was associated with decreased *Candida* burdens in feces. In conclusion, gut *C*. *albicans* worsened *C*. *difficile* infection, possibly through exacerbation of inflammation. Hence, a mouse model of *Clostridium difficile* infection with *C*. *albicans* present in the gut may better model the human patient condition. Gut fungal mycobiome investigation in patients with *C*. *difficile* is warranted and may suggest therapeutic targets.

## Introduction

*Clostridium difficile*, an anaerobic spore-forming gram-positive bacterium, is the most common cause of nosocomial antibiotic-associated diarrhea of patients in long term care facilities world-wide [1, 2]. *C*. *difficile*-associated infection is also the most common hospital-acquired diarrhea worldwide [1]. Gut microbiota alteration due to antibiotic administration diminishes pathogen colonization resistance and is a well-known risk factor for *C*. *difficile* infection [2, 3]. Interestingly, *C*. *difficile*-induced GI leakage is characterized by i) spontaneous bacteremia deriving from the intestinal microbiota [4], ii) fluorescein isothiocyanate-dextran (FITC-dextran) translocation and iii) elevated serum (1→3)-β-D-glucan (BG) without fungemia [5]. The detection of dextran, a carbohydrate molecule that is not absorbed through the intestine, in serum after oral administration, is a standard gut leakage measurement method [6]. BG is a polyglucoside that is a major component of the fungal cell wall [7]. Spontaneous BG elevation in serum, in the absence of invasive fungal disease or iatrogenic contamination, is also an indirect indicator of gut leakage [5, 8]. *C*. *difficile* toxins are responsible for intestinal barrier damage through the disruption of actin cytoskeleton and tight junctions of gut epithelial cells, thus potentiating the translocation of BG [9]. Further, prolonged antibiotic administration, an important risk factor for *C*. *difficile* infection, has been shown to induce the overgrowth of *Candida* spp. (a ubiquitous human GI commensal organism), potentially enhancing the burden of translocatable BG [2, 3, 10, 11]. Although *Candida* overgrowth is well studied in humans, *Candida* spp. are not the predominant fungi in the murine GI tract [12]. Yamaguchi et al. demonstrated that *Candida albicans* in mouse feces was detectable only after a specific mouse chow administration [11]. We also demonstrated that fecal *Candida* is undetectable in mice without oral administration [8, 13]. Interestingly, *Candida* colonization has been demonstrated to alter the course of several mouse models of sepsis and food allergy [8, 13, 14]. Accordingly, *Candida* presence in mouse gut might have an impact on several animal models, especially with respect to intestinal inflammation, and the *Candida* colonization mouse model might more closely resemble human patient conditions. Based upon the role of *C*. *difficile* in enhancing intestinal permeability, and the role of BG as a pro-inflammatory pathogen-associated molecular pattern (PAMP) with pro-inflammatory synergy with other PAMPS [15, 16], we investigated the role of introduced *C*. *albicans*, and derivatives, upon the course and symptom severity of murine *C*. *difficile* infection.

## Materials and methods

### Animals

The animal care and use protocol followed that of the National Institutes of Health (NIH) USA (#85–23, revised 1985). The animal protocols number SST 07/2560 were approved by the Institutional Animal Care and Use Committee of the Faculty of Medicine, Chulalongkorn University, Bangkok, Thailand. Male, C57BL/6 mice at 8-week-old (National Laboratory Animal Center, Nakhornpathom, Thailand) were used. A total of 96 mice were randomized into several groups including 92 mice for the survival studies and experiments and 12 mice for the microbiome analysis.

### Fecal mycobiome analysis

**Fig 1:**
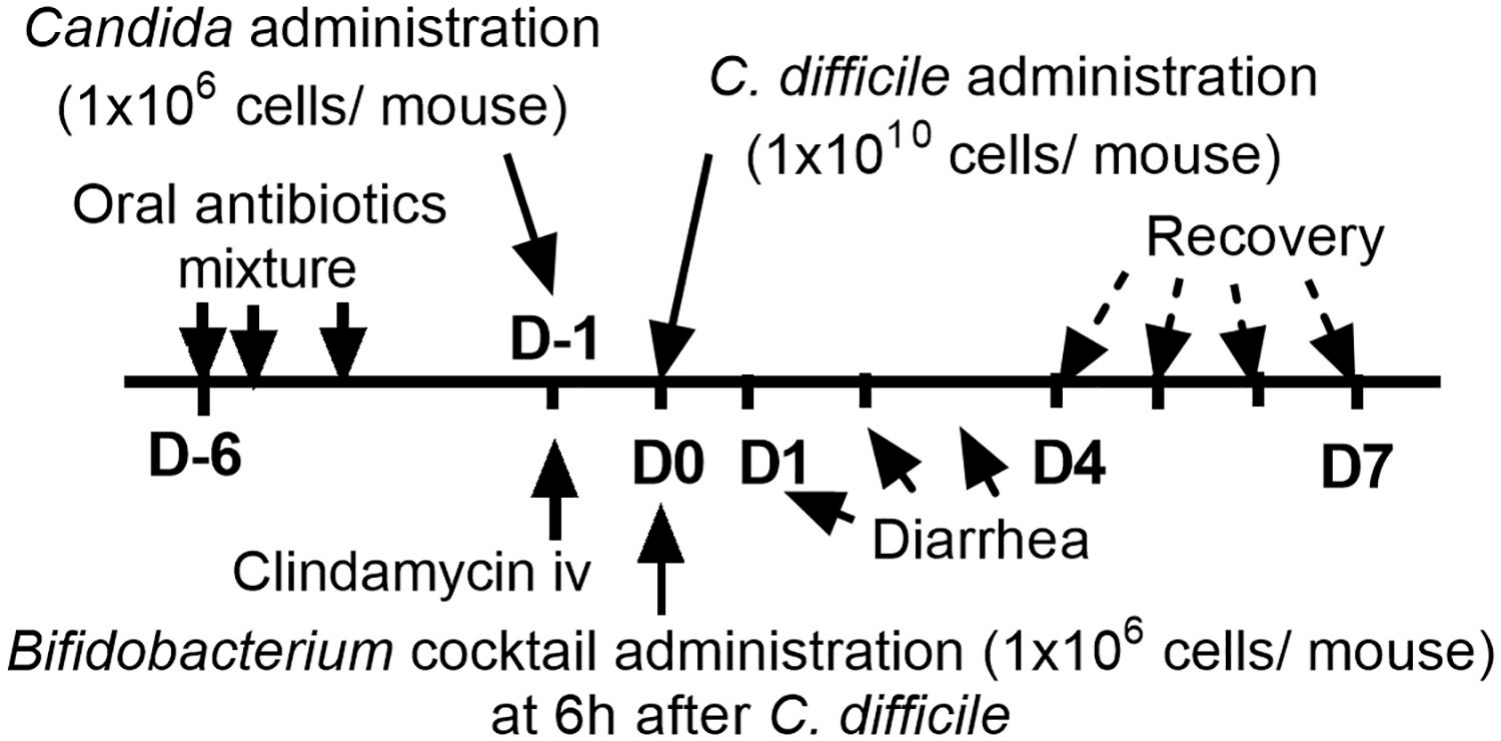
Timeline of the experiment. The timeline of the *C*. *difficile*-infection mouse model is shown. *C*. *difficile*-infected mice developed diarrhea at D1-D3 and were recovered, or dead, after D4.

Because i) the enhancement of gut *Candida* colonization by oral antibiotics in patients is well-known [10], ii) *Candida* spp. are not the predominant fungi in mouse feces [11] and iii) data on fungal colonization in mouse are sparse, we analyzed the mycobiota in feces of antibiotic-administered mice. Antibiotic cocktail (0.5 ml) containing gentamicin (3.5 mg/kg), colistin (4.2mg/kg), metronidazole (21.5mg/kg) and vancomycin (4.5mg/kg) (Sigma Aldrich, St. Louis, MO, USA) was administered twice a day for 3 days (D-6 to D-4) and feces were collected on D-1 (Fig 1). For fecal collection, mice were sacrificed and all visible feces from cecum to rectum were collected and mixed together. Feces from 3 mice (0.25g per mouse) were combined before microbiota analysis. The antibiotic-administered group (6 mice) was divided into ATB#1 and #2 and the control group (6 mice) was divided into control#1 and #2. Of note, mice in the same groups were housed in different cages because co-housing might induce similar gut microbiota within the same cage [17].

Subsequently, feces from each group (0.25g) were processed for metagenomic DNA extractions with 3 independent extractions per sample. Total nucleic acid was extracted using a Power DNA Isolation Kit (MoBio, Carlsbad, CA, USA). Agarose gel electrophoresis and nanodrop spectrophotometry were used to assess metagenomic DNA quality. Universal eukaryotic 1391F (forward, 5'-GTACACACCGCCCGTC-3') and EukBr (reverse, 5'-TGATCCTTCTGCAGGTTCACCTAC-3'), with appended 5' Illumina adapter and 3' Golay barcode sequences, were used for 18S rRNA gene library construction [16]. Each 25-μl PCR reaction comprised 1×EmeraldAmp1 GT PCR Master Mix (TaKaRa), 0.2μM of each primer, and 75 ng of the metagenomic DNA. Independent triplicate polymerase chain reactions (PCRs) were performed and pooled to prevent stochastic PCR bias. The 18S rDNA amplicons of 258 basepairs (bp) were purified from agarose gel, using the GenepHlow^™^ Gel Extraction Kit (Geneaid Biotech Ltd., New Taipei City, Taiwan), and quantified by Picogreen (Invitrogen, Eugene, Oregon, USA). Each sample (240 ng) was pooled for Miseq300 platform sequencing (Illumina, San Diego, CA, USA), using the sequencing primers and index sequence described [17]. Mothur’s standard operating procedures for MiSeq platform were used for the quality screening of the raw sequences [18] then aligned and assigned taxa (operational taxonomic unit, OTU) based on a default parameter [18]. Samples were normalized to an equal sampling depth (N = 118121 reads per sample) following Mothur’s computational method [18].

### *Clostridium difficile* infection mouse model and *Bifidobacterium* cocktail administration

The *Clostridium difficile* mouse model was set up as previously published [5, 18]. Briefly, 0.5 ml of the antibiotic cocktail was orally administered twice a day for 3 days (Fig 1). At Day 2 after last dose of oral antibiotics (D-1) mouse feces were collected for fecal eukaryotic 18S rDNA analysis (mycobiome) (detail above). In another group of these mice, a single dose of clindamycin (10 mg/kg) was administered intra-peritoneally. *Candida albicans* ATCC 90028 (Fisher Scientific, Waltham, MA, USA) was cultured overnight on Sabouraud dextrose broth (SDB) (Thermo Scientific, Hampshire, UK), counted by hemocytometer and orally administered 6h later. To select the dose of the *Candida*-administration, *C*. *albicans* at 1x10^4^-10^10^ CFU were gavaged at D-1. Then mouse feces and blood (through tail vein nicking) were collected daily for 3 days starting at 6h after gavage (D-1) to D1 (see Fig 1) to determine fungi in blood and feces by culture (detail below). For fecal collection, the individual mice were placed in a metabolic cage (Hatteras Instrument, Cary, NC, USA) for a few hours. Then *Candida* at a dose of 1x10^6^ CFU was administered (selected due to positive fecal fungal culture in the absence of candidemia at least 3 days after the administration). Subsequently, 0.5 ml of *C*. *difficile* 1×10^10^ CFU/ml (ATCC BAA1870, Manassas, VA, USA) in NSS or NSS alone was administered orally at D0 (Fig 1). The control group received oral and intra-peritoneal antibiotics but no organism challenge. In addition, a combination of *Bifidobacterium adolescentis*-B24 (BA-B24) and *Bifidobacterium catenulatum*-NB38 (BC-NB38), in a ratio of 1:1 in a total dose at 1x10^6^ cell/ mouse, was orally administered at 6h after *C*. *difficile* gavage, to test the effects of the probiotic (Fig 1).

Humane endpoints were used to euthanize mice in distressed condition. The criteria for euthanasia included inability to maintain standing position, hunched position, agonal breathing, weight loss more than 15% and impaired mobility after a stimulation [19]. Mice were monitored daily and mice meeting the criteria were euthanized by cardiac puncture under isoflurane anesthesia. All mice were sacrificed at 7 days post-*C*. *difficile* infection.

### Gut leakage measurement

Intestinal epithelial permeability (gut leakage), in the model, was determined by serum fluorescein isothiocyanate-dextran (FITC-dextran) and elevation of serum BG without fungemia, as described previously [5, 20]. Briefly, FITC-dextran (molecular weight 4.4 kDa, Sigma, St. Louis, MO, USA) at 0.1 ml/ gram (of 25 mg/ml solution) was orally gavaged at D1 (24h after *C*. *difficile* administration) and blood was collected 3h later, through cardiac puncture, under isoflurane, for serum FITC-dextran detection. Blood was allowed to clot, centrifuged, and serum was kept at -80°C until analyzed. Serum BG was analyzed with Fungitell (Associates of Cape Cod, Inc., East Falmouth, MA, USA) and values beyond the lower and upper range of the standard curve at <31 and >500 pg/ml were recorded as 0 and 523 pg/ml, respectively. Serum FITC-dextran was measured by the fluorospectrometry (NanoDrop 3300; Thermo Scientific, Wilmington, DE, USA) with the excitation and emission wavelength at 485 and 523 nm, respectively, against a standard curve of serially diluted FITC-dextran in phosphate buffer solution (PBS).

### Mouse sample analysis

For quantitative bacterial analysis, blood (25μl) was spread directly onto blood agar plates (Oxoid, Hampshire, UK), incubated at 37°C and bacterial colonies were enumerated at 24-48h. In parallel, macrophage inflammatory protein 2 (MIP-2), keratinocyte chemoattractant (KC), tumor necrosis factor (TNF)-α and interleukin (IL)-1β, the pathogenesis associated cytokines of *C*. *difficile* infection [2], were measured in serum with ELISA assays (PeproTech, NJ, USA). All assays were performed according to the manufacturer’s protocol. For the fungal burden in blood, blood (50 μl), in serial dilution, was directly plated on Sabouraud Dextrose Agar (SDA) with 0.1% chloramphenicol (Thermo Scientific, Hampshire, UK) and Sabouraud Dextrose Broth (SDB) with 0.05 g/l chloramphenicol (Thermo Scientific), then kept at 35°C for 72h before *Candida* colony enumeration. In addition, the colons of the mice were collected at sacrifice, weighed, homogenized with PBS and used for tissue cytokine analysis (MIP-2, KC, TNF-α and IL-1β) as biomarkers of local inflammatory responses.

### Fecal collection and analysis

At sacrifice, all visible feces from cecum to rectum were mixed with PBS in a ratio of 1μg/ 1μl before plating directly onto SDA with 0.1% chloramphenicol (Thermo Scientific) to determine fecal fungal burdens. The plates were incubated at 35°C, for 72h, before fungal colony enumeration. In parallel, fecal fungal identification by polymerase chain reaction (PCR) was also performed. Genomic DNA was extracted from the sample by High Pure PCR Template Preparation Kit (Roche, USA) and quantified by NanoDrop^™^ 1000 Spectrophotometer (Thermo Scientific). The DNA was amplified in the internal transcribed spacers (ITS1 and ITS2) and fungal taxa were identified by the 5.8S rRNA region of fungal universal primer (kit1 and kit4 *i-Taq*^™^ DNA polymerase; iNtRON Biotechnology, Korea).

In addition, the fecal burdens of *C*. *difficile* in the mice were quantified using quantitative real-time PCR. The *C*. *difficile* toxin B (*tcd*B)-specific primers *tcd*B-F [5'-GG AAAAGAGAATGGTTTTATTAA-3'] and *tcd*B-R [5'-ATCTTTAGTTATAA CTTTGACATCTTT-3'] were from the conserved 5 region of *tcd*B and generated a 160-bp fragment, as previously described [21]. The standard curve was created by the LightCycler software using 10-fold serial dilution (1 copy number −10^9^ copies number) per 5 μl of plasmid. The profiling standard curve was indicated as a graph of crossing point (Cp) vs. bacterial copy number. The PCR reaction was performed in a 20 μl mixture containing 4 μl of Faststart DNA LightCycler^®^FastStart DNA Master^PLUS^SYBR Green I (Roche, Germany), 9 μl of nuclease-free water, 1 μl of each 10μM primer, and 5 μl of plasmid template. The PCR reaction was performed as follows: 45 cycles of 95°C for 10s, 55°C for 10s, 72°C for 25s with LightCycler^®^ 2.0 instrument (Roche, Germany). The amplified product was measured by a SYBR green fluorescent signal using LightCycler^®^ FastStart DNA Master^PLUS^SYBR Green I (Roche, Germany). *C*. *difficile* quantification was calculated using the standard curve and shown as bacterial copy number.

### Induction of IL-8 production from human intestinal epithelial cells and dectin-1 inhibition

Human colorectal adenocarcinoma cells from the American Type Culture Collection (Manassas, VA, USA) (HT-29 and Caco-2; ATCC HTB-38 and ATCC HTB-37, respectively) were maintained in McCoy’s 5a modified medium supplemented with 10% (v/v) heat-inactivated FBS and Dulbecco’s Modified Eagle’s Medium (DMEM) supplemented with 20% (v/v) heat-inactivated FBS, respectively at 37°C under 5% CO_2_ and sub-cultured before performing the co-culture assay. For sub-culture, 5x10^4^ cells/well were plated in a 96-well plate. A preparation of heat-killed *C*. *albicans* was used as representative of fungal molecule exposure following a previously published protocol with some modifications [8]. Briefly, *C*. *albicans* at 5x10^5^ or 1x10^6^ cells/ ml (intestinal cells: *C*. *albicans*: ratio at 1:5 and 1:10, respectively) were killed by heating at 65°C for 30 minutes and sonication (the power setting: pulse on 20 Sec with Amplitude 40%, pulse off 5 Sec, and total processing time 60 min) with a High Intensity Ultrasonic Processor (VC/VCX 130, 500,750). Additionally, live *C*. *difficile* in the ratio of 100:1 (bacteria: intestinal cells) were incubated with the cells. The total volume was adjusted by PBS addition to 200 μl/ well. Supernatant was collected at 24h of incubation and cytokines were measured by ELISA (R&D Systems, Minneapolis, MN). Because lipopolysaccharide (LPS) and (1→3)-β-D-glucan (BG) are major cell wall molecules of gram negative bacteria and fungi, respectively, both components were incubated with the intestinal cells to further explore combinatorial effects. Hence, LPS (*Escherichia coli* 026:B6; Sigma-Aldrich) at 100 ng/ml and/ or BG (Pachyman, Megazyme, Bray, Ireland) at 1 μg/ml, alone and in combination, were incubated with the intestinal cells, followed the above-described procedure. In addition, we hypothesized that a synergistic response of intestinal cells against *C*. *difficile* and *Candida* might, at least in part, go through dectin-1 (a potent receptor of BG found on immune system lineage cells). Accordingly, a dectin-1 blocker, 1, 3/1, 6-β-glucan from *Saccharomyces cerevisiae*, a competitive inhibitor of dectin-1 (WGP Soluble (tlrl-wgps), Invivogen, San Diego, CA, USA), at 1 μg/ml was incubated for 1 h before certain of the above-described cell stimulation experiments.

Because of the anti-inflammatory property of conditioned medium from some probiotics [22, 23], we tested this potential effect of *Bifidobacterium* spp. against *C*. *difficile*. *Bifidobacterium*-conditioned media (BCM) were prepared as previously described with modification [24]. Briefly, *Bifidobacterium adolescentis*-B24 (BA-B24) and *Bifidobacterium catenulatum*-NB38 (BC-NB38), inoculated at an optical density of 0.1 at 600 nanometers (OD600) was incubated anaerobically for 48h. Supernatants were collected by centrifugation at 4000 xg for 10 min at 4°C, filtered using a 0.22 μm membrane filter (Minisart, Sartorius Stedim Biotech GmbH, Goettingen, Germany) and 500 μL of supernatants were concentrated by speed vacuum drying at 40°C for 3h (Savant instruments, Farmingdale, NY). Residues, concentrated to near dryness, were re-suspended in an equal volume of McCoy’s 5a modified medium or DMEM medium as *Bifidobacterium* culture media (BCM) and stored at -20°C until further use.

HT-29 and Caco-2 cells (5.0 x 10^4^ cells/well) were pre-cultured in 96 wells plate for 24h. The culture supernatant was replaced with fresh medium containing either (5% vol/vol) BCM of BA-B24 or BC-NB38. Then, *C*. *difficile* were added, at the ratio of bacteria: intestinal cells of 100:1, and the mixed cultures were incubated with and without BCM for 24h. IL-8 concentrations in culture supernatants were determined using the Quantikine Human IL-8 Immunoassay Kit (R&D Systems, Minneapolis, MN).

### Statistical analysis

Mean ±standard error (SE) was used for the data presentation and the differences between groups were examined for statistical significance by one-way analysis of variance (ANOVA) followed by Tukey’s test for the comparisons of multiple groups. Survival analysis and time course analysis were performed by log-rank test with Bonferroni correction and repeated measure ANOVA, respectively. All statistical analyses were performed with SPSS 11.5 software (SPSS, IL, USA). A P-value < 0.05 was considered to be statistically significant.

## Results

### Oral antibiotics enhanced fungal colonization in mouse gut; A mycobiota analysis

**Fig 2:**
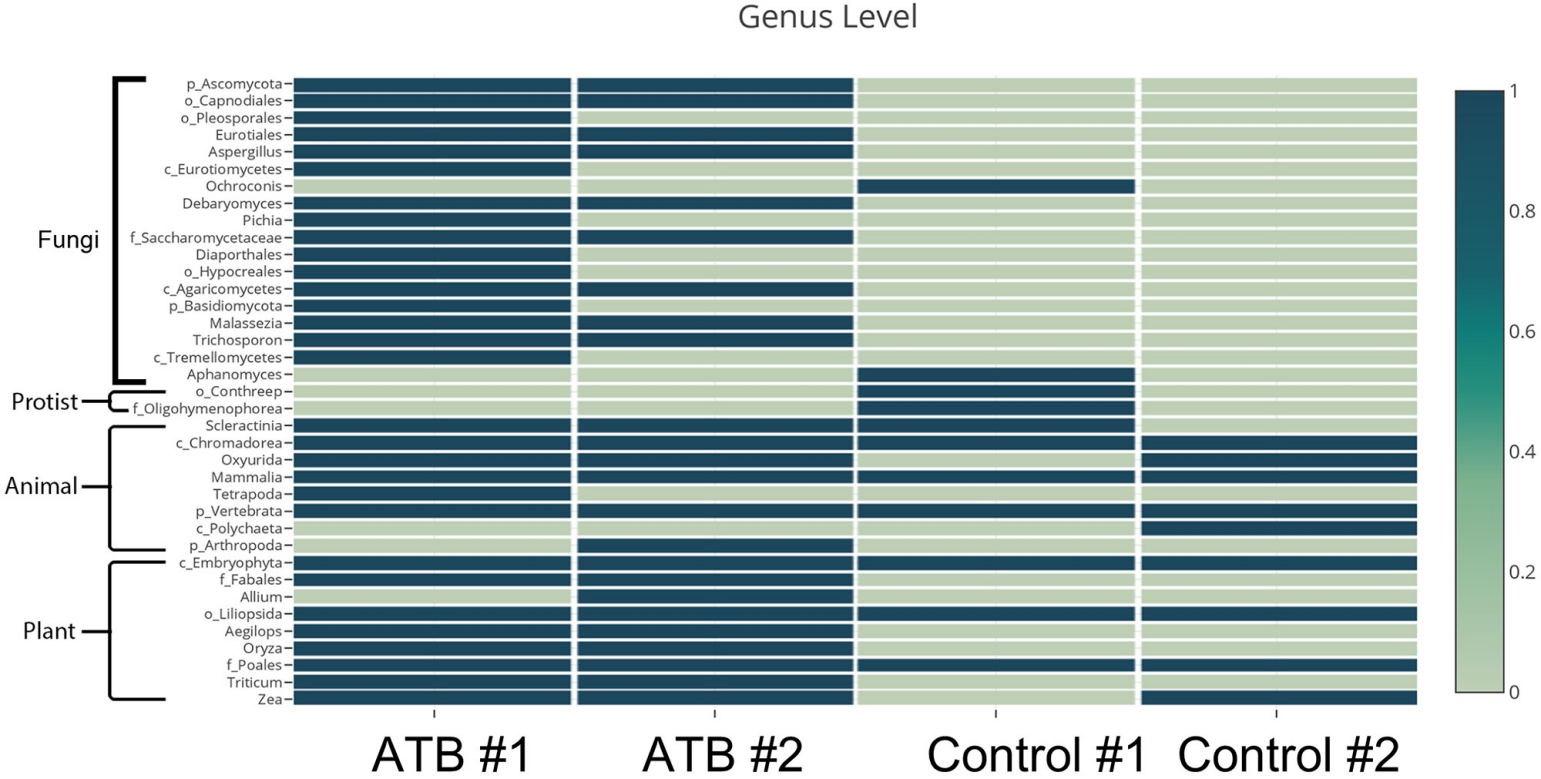
Fecal microbiome analysis. The 18S rDNA analysis of mouse feces, with and without antibiotic administration (ATB and Control, respectively) was analyzed (combined feces from 3 mice in each group).

Conventional culture and polymerase chain reaction (PCR) for fungal identification were performed upon mouse feces after the oral antibiotic cocktail (at D1 of the model; Fig 1). These were negative in all mice (data not shown). Surprisingly, the 18S rDNA analysis revealed several groups of fungi after antibiotic administration but very few in the control group suggesting increased sensitivity of 18S rDNA analysis over regular PCR. Fungi, in the phyla *Ascomycota* (eg. *Aspergillus* and *Pichia*) and *Basidiomycota* (eg. *Trichosporon*) were presented in mouse feces after antibiotic treatment but not in control (Fig 2). Increased 18S rDNA of plant and animal origin was observed in the antibiotic-treated mice, presumably due incomplete digestion of chow. In contrast, *Candida* spp. in human feces is easily demonstrated by culture (data not shown). Because the scarcity of *C*. *albicans* in mouse gut compared with human might influence the mouse model illness severity, we administered *C*. *albicans* in the *Clostridium difficile* model.

### *Candida albicans* administration enhanced the severity of the *Clostridium difficile* infection model

**Fig 3:**
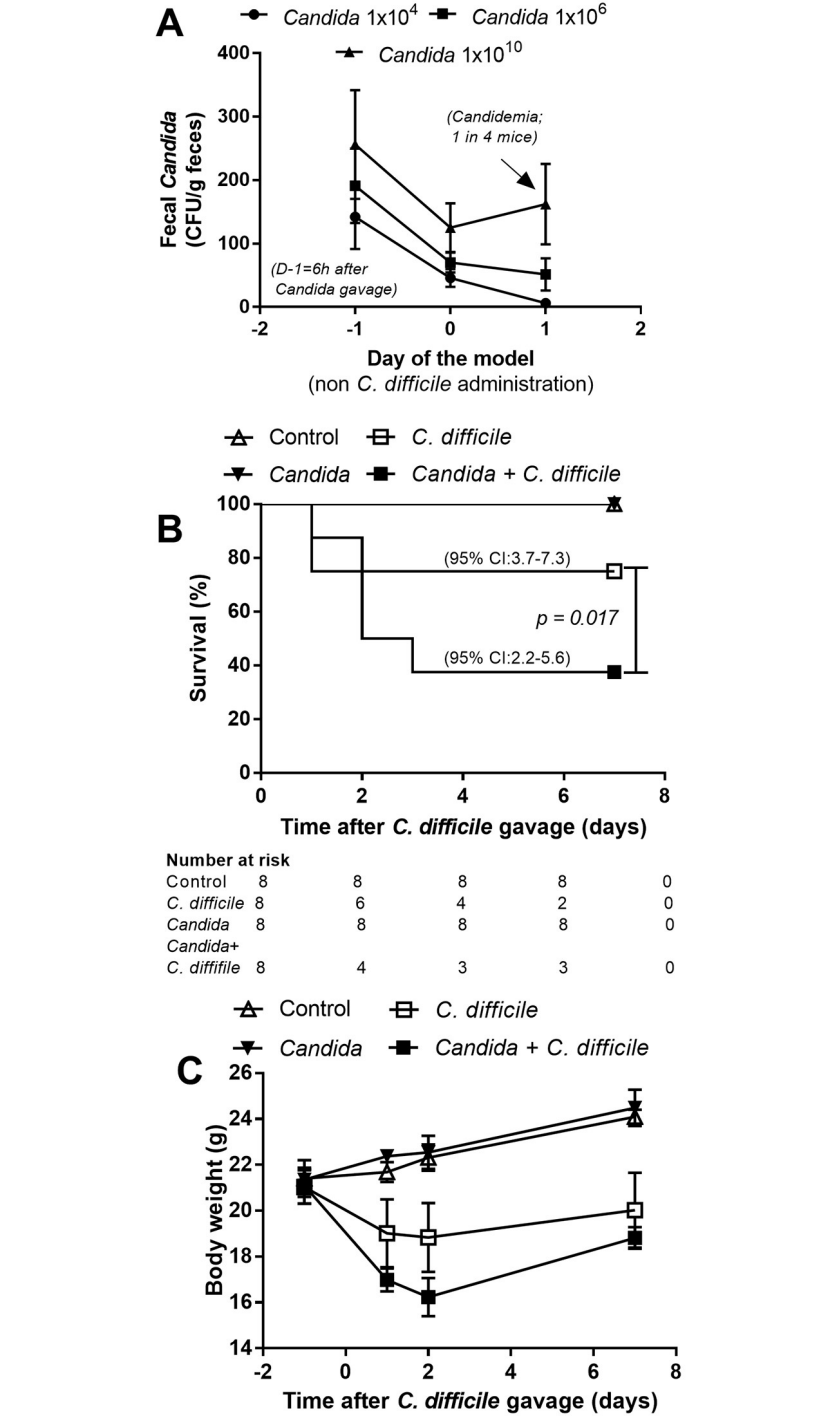
*Candida albicans* dose determination. To determine the appropriate dose of *C*. *albicans*, fecal fungi and fungemia were analyzed after the administration of *C*. *albicans* at several dose levels, without *C*. *difficile* gavage (n = 4/group) (A). The severity of the *C*. *difficile*-infection mouse model and control (non-*C*. *difficile*) with and without *C*. *albicans* administration (1x10^6^ CFU) as determined by survival analysis (n = 8/group) (B) and weight loss (n = 6-8/time point, except for D7 of *Candida* + *C*. *difficile* group; n = 3) (C) are shown. 95%CI, 95% confidence interval.

The dose of administered *Candida* could have a variable impact upon the natural course of *C*. *difficile* infection. Accordingly, to optimize, we orally administered *C*. *albicans* in several doses (1x10^4^-1x10^10^ CFU) and measured fungi in feces and blood. Recoverable *Candida* was present in feces as early as 6h after the oral administration, but without significant differences in fungal burdens among the dosage groups (Fig 3A). However, fecal fungi were undetectable in 3 of 4 mice, with a dose of 1x10^4^ CFU, and spontaneous candidemia was demonstrated in 1 of 4 mice at 3 days after fungal administration at a dose of 1x10^10^ CFU/mouse (Fig 3A). Accordingly, *C*. *albicans* dosing at 1x10^6^ CFU was selected for use in all further experiments based upon its providing the most consistent fecal fungi levels, absent candidemia, in comparison with other doses.

**Fig 4:**
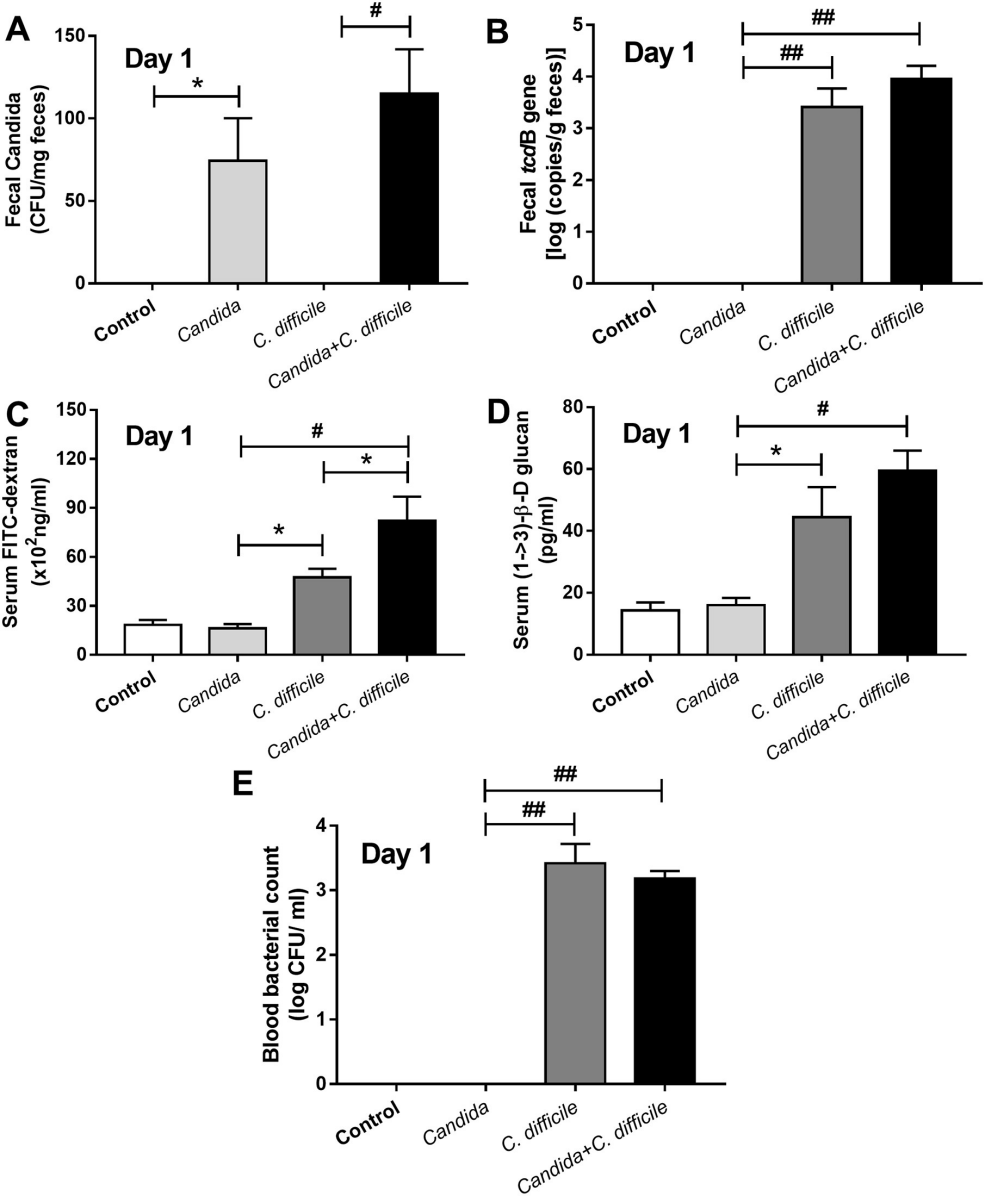
Impact of *Candida* gavage upon *Clostridium difficile* model. To determine the impact of gavaged *Candida* in the *C*. *difficile* mouse model, fecal *Candida* (A), fecal *C*. *difficile* toxin B (B), gut leakage by FITC-dextran assay and serum (1→3)-β-D-glucan (BG) (C, D) and bacteremia (E), were measured (n = 6-8/group). *, p<0.05; #, p< 0.01; ##, p< 0.001.

**Fig 5:**
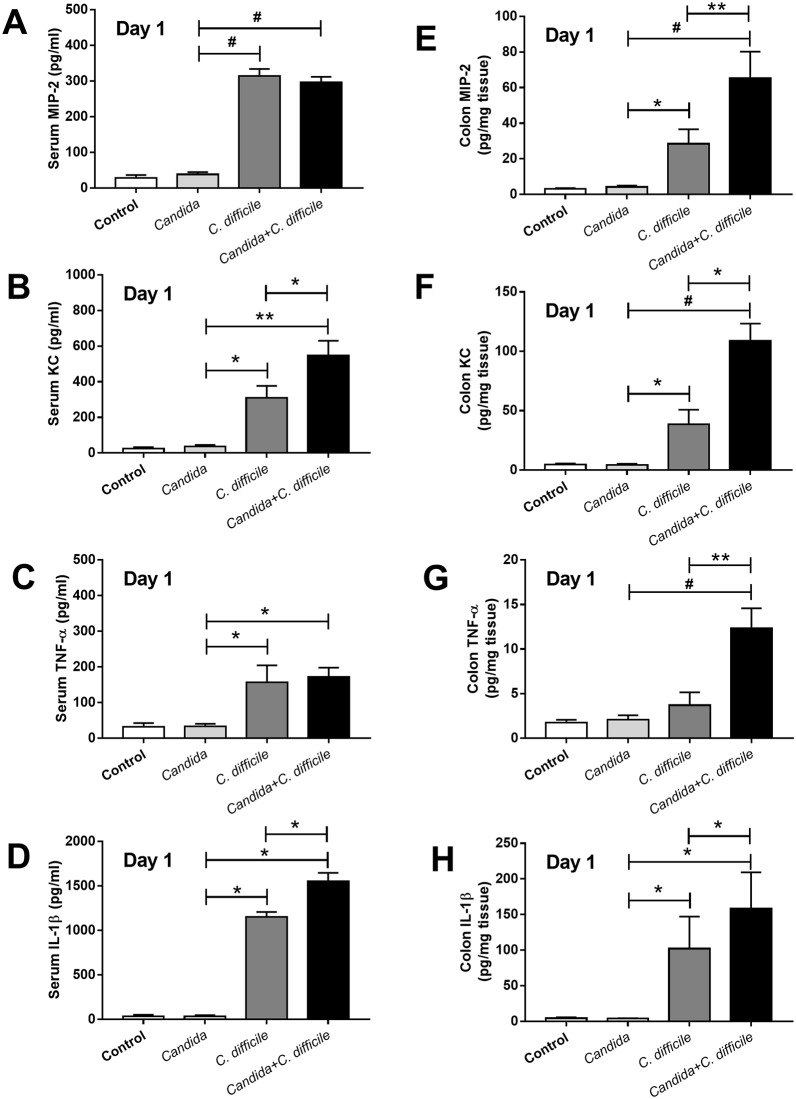
Inflammatory responses in the model. The systemic and local inflammatory responses as determined by several inflammatory cytokines in serum and colon tissue, respectively, in *C*. *difficile*-infection mouse model and control (non-*C*. *difficile*) with and without *C*. *albicans* administration are presented (n = 6-8/group) (A-H). *, p<0.05; **, p<0.01; #, p<0.001; MIP-2, macrophage inflammatory protein 2; KC, keratinocyte chemoattractant; TNF-α, tumor necrosis factor-α; IL-1β, interleukin-1β.

The *C*. *difficile* model with *Candida* administration, as compared with *C*. *difficile* gavage alone, was more severe as demonstrated by i) reduced survival rate (from 75% into 38%, respectively) and ii) enhanced generalized bowel edema, 75% of *C*. *difficile* +*Candida* mice and 25% of *C*. *difficile* mice, respectively (Fig 3B and S1 Fig), but without a difference in weight loss (Fig 3C). D7 weight was not compared due to the marked mortality difference between the groups. In addition, no direct impact of co-exposure upon the respective populations of *C*. *albican*s and *C*. *difficile* was observed in the model. Neither fecal *Candida* nor microbial burden (*Clostridium* toxin B gene burden) in feces was increased with the co-administration (Fig 4A and 4B). Although *Candida* alone did not cause gut leakage, *Candida* enhanced *C difficile* induced gut leakage as determined by FITC-dextran assay but not by serum BG and bacteremia (Fig 4C–4E). In addition, culturable candidemia was undetectable in all mice (data not shown). In addition, in comparison with *C*. *difficile* infection alone, the *Candida* + *C*. *difficile* model showed an increase in only some serum cytokines (serum KC and IL-1β) (Fig 5A–5D). In contrast, a broader spectrum of increased, but localized, inflammatory cytokines (MIP-2, KC, TNF-α and IL-1β) appeared in colon tissue (Fig 5E–5H).

### *Candida albicans* induced pro-inflammatory cytokine production in human intestinal cell-lines

**Fig 6:**
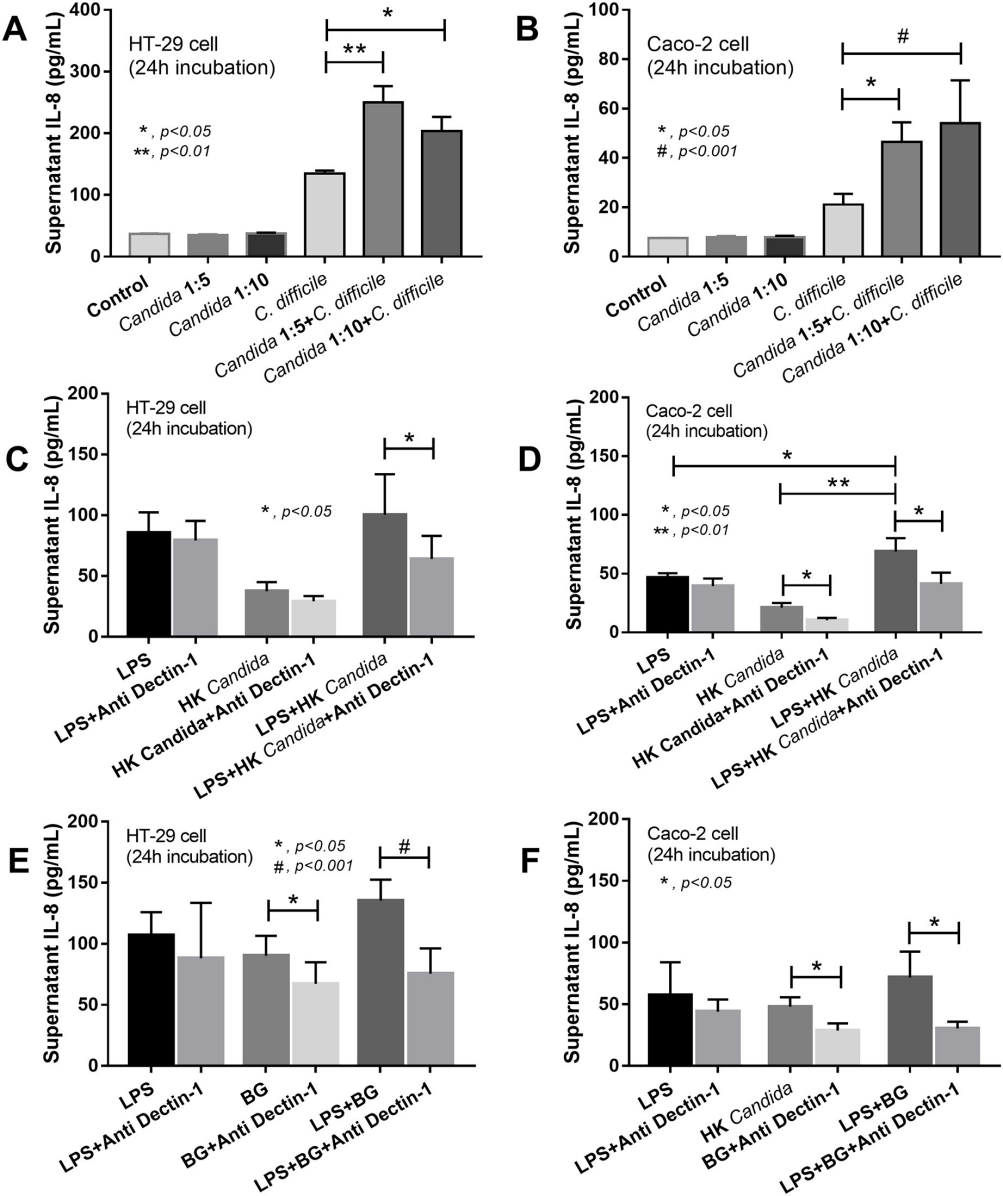
Stimulations of HT-29 and Caco-2 cell-lines. IL-8 cytokine levels in the culture supernatants of human intestinal epithelial cell lines (HT-29 and Caco-2 cell) after activation by *C*. *difficile* or phosphate buffer solution (PBS) as control, with and without the cell lysate of heat-killed *C*. *albicans* at the ratio of intestinal cells: *C*. *albicans* at 1:5 or 1:10 (see Method) are presented (A, B). In addition, lipopolysaccharide (LPS) alone or with heat-killed *C*. *albicans* or (1→3)-β-D-glucan (BG), with and without dectin-1 blocker, were used toward HT-29 and Caco-2 cells (C-F) (independent experiments were performed in triplicate) *, p<0.05; **, p<0.01; #, p<0.001.

As intestinal epithelial inflammation is one of the important mechanisms responsible for gut leakage, we investigated whether the cell lysate of *C*. *albicans* could induce IL-8, an important pro-inflammatory chemokine [25]. *Candida* lysate together with *C*. *difficile*, but not *Candida* lysate alone, strongly enhanced IL-8 production from the intestinal cells compared with *C*. *difficile* alone. However, the enhancement by *Candida* lysate with *C*. *difficile* was not dose-dependent, in the range tested (Fig 6A and 6B). The *Candida* enhancement of *C*. *difficile*-related inflammatory stimulus might reflect cell wall beta-glucan (BG) interaction with the dectin-1 receptor in the host or on cells in culture [26]). The response to co-stimulation with LPS and heat-killed *Candida* declined with the addition of dectin-1 beta-glucan. However, only *Candida*-related stimulation (Caco-2 cell only), but not LPS stimulation, was inhibited (Fig 6C and 6D). There was an additive response as demonstrated by higher supernatant IL-8 in LPS + *Candida* than with LPS alone in Caco-2 cells (Fig 6D). Likewise, in LPS with BG incubation, dectin-1 blocker again reduced the intensity of the co-stimulation and attenuated the reaction of BG alone (but not the reaction of LPS alone) (Fig 6E and 6F). There was no additive response with regard to IL-8 elicitation with LPS+BG compared to LPS stimulus alone (Fig 6E and 6F).

### *Bifidobacterium* cocktail effectively attenuated the *C*. *difficile* plus *Candida* model severity

**Fig 7:**
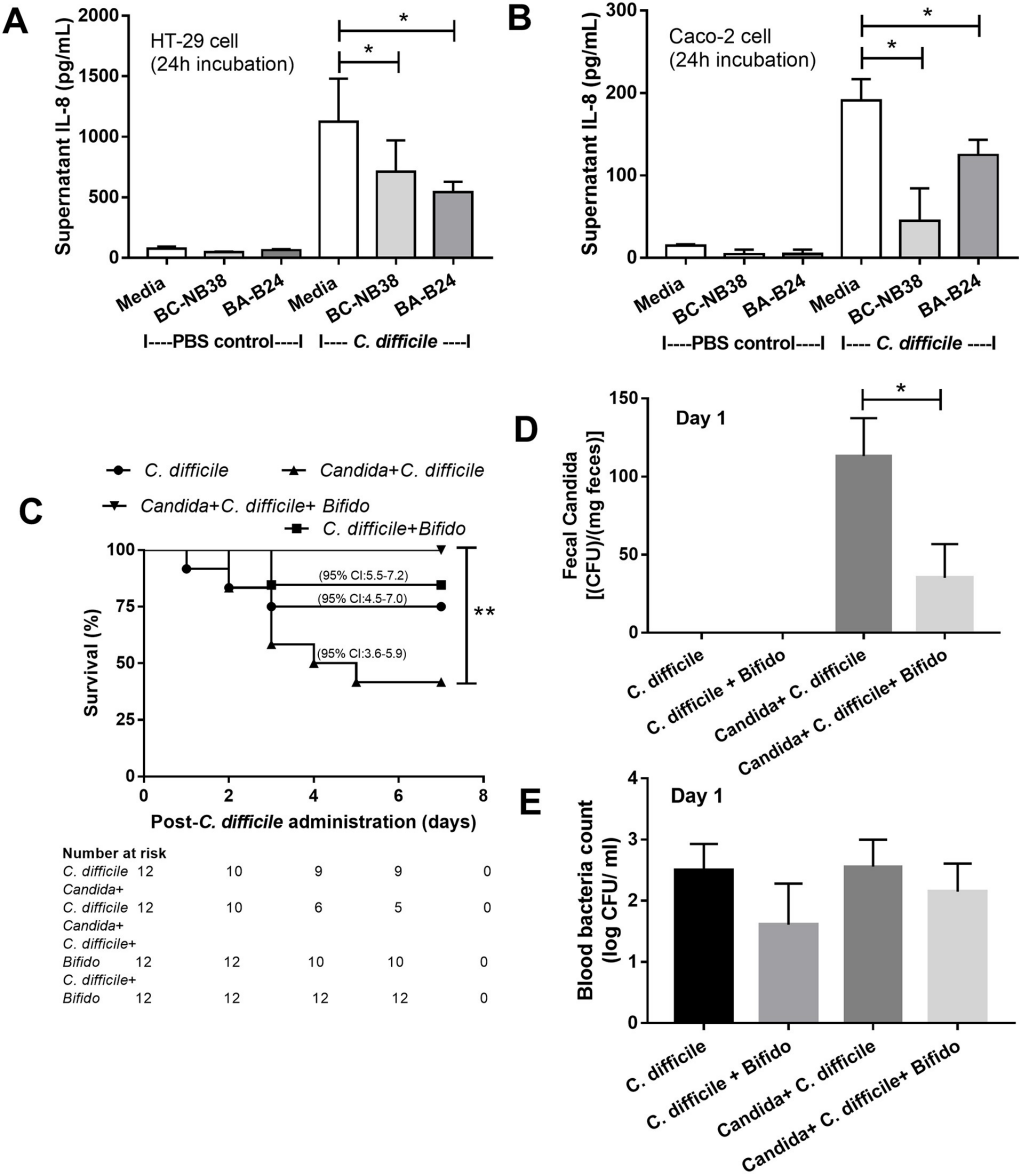
The *in vitro* effect of the probiotic. IL-8 cytokine levels in the supernatants of human intestinal epithelial cell lines (HT-29 and Caco-2 cell) after activation by *C*. *difficile* or phosphate buffer solution (PBS) control, with and without *Bifidobacterium adolescentis*-B24 (BA-B24) and *Bifidobacterium catenulatum*-NB38 (BC-NB38), are presented (A, B) (independent experiments were done in triplicate); Survival analysis (C) (n = 12/ group); fecal fungal burdens (D); and blood bacterial count (E) were measured to determine the effect of *Bifidobacterium* cocktail (Bifido; see Methods) against *C*. *difficile* with and without *C*. *albicans* administration (n = 5-7/group for D-E). *, p<0.05; **, p< 0.02.

**Fig 8:**
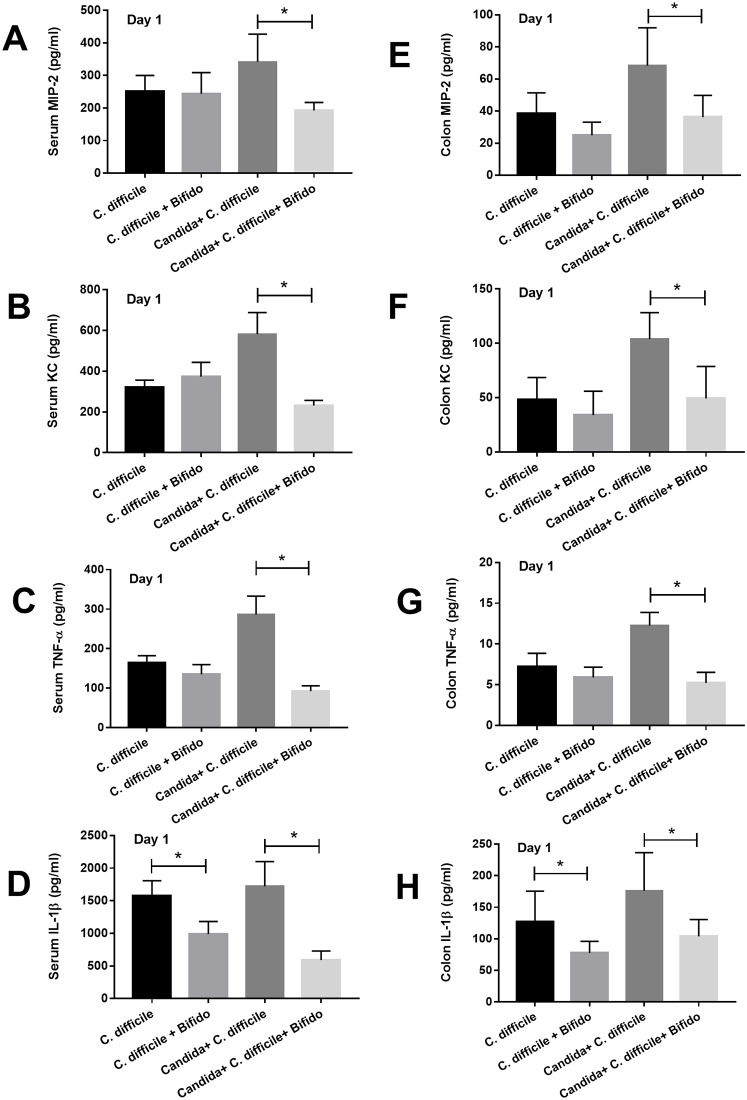
The *in vivo* effect of the probiotic. The systemic and local inflammatory responses as determined by several inflammatory cytokines in serum and colon, respectively, after the administration of *Bifidobacterium* cocktail (Bifido; see Method) in *C*. *difficile*-infection with and without *C*. *albicans* are presented (n = 6-8/group) (A-H). *, p<0.05; MIP-2, macrophage inflammatory protein 2; KC, keratinocyte chemoattractant; TNF-α, tumor necrosis factor-α; IL-1β, interleukin-1β.

We tested the effectiveness of *Bifidobacterium* both *in vitro* with the intestinal cell lines and *in vivo*, in the two models. First, the cytokine elicitation attenuation properties of the conditioned media from *Bifidobacterium adolescentis*-B24 (BA-B24) and *Bifidobacterium catenulatum*-NB38 (BC-NB38) were demonstrated by the decrease in IL-8 production of the *C*. *difficile*-activated intestinal cells (HT-29 and Caco-2) (Fig 7A and 7B). Second, the administration of both strains in combination (*Bifidobacterium* cocktail) improved the survival rate in the *C*. *difficile* model with *Candida* but not the model without *Candida* (Fig 7C). Likewise, the cocktail significantly attenuated fecal fungal burdens, but not blood bacterial burdens (Fig 7D and 7E). The probiotics also attenuated all cytokines (MIP-2, KC, TNF-α and IL-1β) either in serum or colon in *C*. *difficile* model with *Candida* but reduced only IL-1β (in serum and colon) in the model without *Candida* (Fig 8A–8H).

## Discussion

*Candida albicans* is not a prevalent gastrointestinal microorganism in mouse. Oral challenge with *Candida* exacerbated several markers used to characterize the severity of the course of a *C*. *difficile* infection in a murine model. Oral-administration of *C*. *albicans* prior to *C*. *difficile* administration enhanced the disease severity through increased gut inflammation. Given that gut *Candida* spp. are commensal organisms in humans and are observed to experience large increases in number under the influence of a wide variety of antibiotics, the murine model of *C*. *difficile* with *C*. *albicans* might more closely resemble human conditions.

Although antibiotic-induced bacterial dysbiosis in gut is well-known [27–29], the impact of antibiotics upon gut fungi is still an emerging area of study [30]. Mouse gut fungi are undetectable by conventional mouse feces culture [8, 11, 13] but are easily identified in human [10, 31]. Antibiotic effects upon gut levels of *Ascomycota* (*Candida*, *Aspergillus* and *Pichia* [32, 33]), *Basidiomycota* (*Tricosporon* [34]), and *Ochroconis* and *Aphanomyces*, a dematiaceous fungus and a water mold [35, 36], respectively, have been observed. *Pichia*, a group with some species re-classified as *Candida* [33, 37], were detectable after antibiotic administration despite being undetectable in normal controls.

It is important to consider the role of intestinal fungi in models of gut inflammation as fungi are present in humans and they may be beneficial or detrimental. While gut fungi attenuate the severity of colitis in dextran sulfate solution model possibly through competition with gut pathogenic bacteria [38], *C*. *albicans* in gut worsen sepsis severity [8, 13]. This may occur, in part, through increased gut permeability as evidenced by translocation of BG, a major molecular of fungal cell wall [8, 13]. Because of the low abundance of *C*. *albicans* in mouse gut, a *C*. *difficile* murine model with *C*. *albicans* might be a better representative translational model.

Here, we demonstrated that an optimal dose of *C*. *albicans*, enough to be detectable in feces by culture for a few days (but in the absence of candidemia), increased the severity of the *C*. *difficile* infection model. This may be partly due to increased gut-pathogen burdens (*C*. *albicans* + *C*. *difficile*) that directly amplified enterocyte inflammation and enhanced gut-translocation of pathogens, PAMPs, or other microbially-derived moeities. *Candida* gavage alone did not induce gut leakage (as indicated by lack of translocation of FITC-dextran, serum BG and bacteremia) and did not amplify the burdens of *C*. *difficile* in gut. In contrast, *C*. *difficile* is a well-known organism that directly degrades gut tight-junctions [5, 39]. Tellingly, *Candida* administration along with *C*. *difficile*, in comparison with *C*. *difficile* alone, enhanced gut leakage as determined by FITC-dextran (molecular weight; MW 4.4 kDa) although the leakage possibly did not permit larger molecules such as BG (MW 30–300 kDa) to translocate [40]). In addition, co-exposure to *Candida* and *C*. *difficile* increased local inflammatory responses as demonstrated by more elevated cytokine levels from gut specimens and corroborated by *in vitro* experiments on intestinal cell-lines. *Candida* or BG (a major fungal molecule) also enhanced the response of LPS against the intestinal cell-lines, presumably through dectin-1 activation. Dectin-1 blockage neutralized this effect. Thus, the *in vitro* observations suggest that introduction of *Candida* into the gut enhances the enterocyte response towards both *C*. *difficile* and, potentially, various gut-predominant Gram-negative bacteria, in part, through BG-related inflammatory responses. These data support the conclusion that the *C*. *difficile* mouse model with *Candida* is different from the conventional model with *C*. *difficile* alone.

To assess potential translational utility of this model, *Bifidobacterium* were tested for a probiotic effect. Bifidobacterium attenuated the severity of the *C*. *difficile* with *Candida* model demonstrating improved survival. However, only a limited effectiveness in the model without *Candida* was observed. The probiotic attenuated the *C*. *difficile* + *Candida* model through decreased fecal fungal burdens, but not reduced clostridium toxin in feces, suggesting that a therapeutic strategy focusing on fungal reduction might be useful in *C*. *difficile* infection. Gut-derived BG-induced hepatic inflammation has also been observed [41]. Additional studies of fungal burden characterization in *C*. *difficile* infection as well as gut fungal burden mitigation are warranted.

## Conclusions

The presence of *C*. *albicans* in gut enhanced the severity of a murine *C*. *difficile* infection model, through the enhancement of intestinal inflammation. This work informs pre-clinical model development for the study of *C*. *difficile* infection and suggests translational research approaches.

## Supporting information

S1 Fig Representative intestinal appearance in mice of the control and *Candida albicans* administration groups (A-B), localized intestinal edema (only at large bowel) from *Clostridium difficile* gavage alone (C) and the generalized intestinal edema (both large and small bowel) in *C*. *albicans* plus *C*. *difficile* administered mice (D) are presented. (TIF)
