# Supplementary figures and images for: Oral Candida administration in a Clostridium difficile mouse model worsens disease severity but is attenuated by Bifidobacterium
Source: PLoS One. 2019 Jan 15;14(1):e0210798. doi: 10.1371/journal.pone.0210798 (PMC6333342; doi:10.1371/journal.pone.0210798)

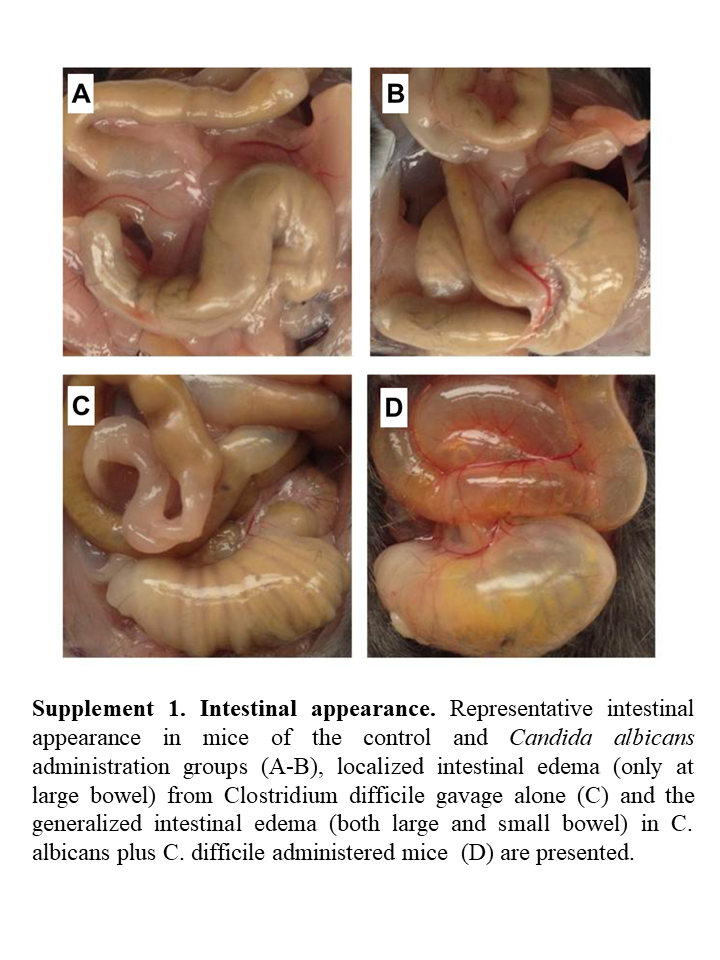

Supplement: S1 Fig — (TIF) [file pone.0210798.s001.tif]
